# Uric Acid and the Heart

**DOI:** 10.1371/journal.pmed.0020090

**Published:** 2005-03-29

What is the level of medical evidence that should be used to inform medical practice? At the bottom of the hierarchy of evidence are anecdotes, expert opinion, case reports, and case series, and at the top is the systematic review of published (and sometimes unpublished) evidence. By necessity, systematic reviews come many years after hypotheses are first raised, and in the interim recommendations for practice may sway back and forth. One example of this is the debate over the role of uric acid in heart disease, which has been going on for more than 50 years. It started with a paper published in 1951 in the *Annals of Internal Medicine* that found higher serum uric acid concentrations in patients with coronary heart disease (CHD) compared with controls. Since then, measurement of serum uric acid has been suggested as a predictor of CHD. But many of the studies on serum uric acid are epidemiologic studies—somewhere in the middle of the hierarchy of evidence—and have come to different conclusions about how useful measurement of uric acid is.

Dissecting out the role of uric acid is further complicated by three things: high levels of uric acid are associated with hypertension and being overweight (other risk factors for CHD); levels of uric acid can be altered by drugs such as diuretics that people with CHD often take; and finally, alteration of renal function can affect uric acid levels. Another problem is the type of studies that have been used to address the question of uric acid's role in CHD. Retrospective studies may be unable to control adequately for other risk factors—hence prospective, ideally population-based, studies would be the best to answer the question of whether there really is an association between high uric acid and CHD.

In this month's *PLoS Medicine*, John Danesh and colleagues from the University of Cambridge, along with investigators from the Icelandic Heart Association, report the single largest prospective study addressing the role of uric acid in heart disease. Further, their systematic review combines their findings with those of 15 previously published prospective studies of serum uric acid—9,458 cases of CHD and 155,084 controls in all.

The paper answers the question of the role of uric acid in prediction of CHD clearly: the risk ratio for prediction of disease was 1.13 (1.07–1.20), but it was only 1.02 (0.91–1.14) in the eight studies that had the most complete adjustment for possible confounders. What this paper does not do is directly address the question of whether or not serum uric acid is involved in causing CHD through intermediates; however, it does suggest that serum uric acid levels are unlikely to be a major determinant of CHD.

Where does such a result leave patients? Well, it is likely that improving diet, losing weight, and controlling blood pressure may all contribute to reducing both one's risk of CHD and one's serum levels of uric acid. The role of uric acid in CHD is now likely to be of interest only to those studying basic science; for now, the clinical question seems closed.

